# A new model to predict soil thermal conductivity

**DOI:** 10.1038/s41598-023-37413-5

**Published:** 2023-07-01

**Authors:** Kun Xiong, Yuqing Feng, Hua Jin, Sihai Liang, Kaining Yu, Xingxing Kuang, Li Wan

**Affiliations:** 1 School of Water Resources and Environment, China University of Geosciences (Beijing), Xueyuan Road 29, Beijing, 100083 China; 2 Hebei Center for Ecological and Environmental Geology Research, Hebei GEO University, Shijiazhuang, 050031 China; 3 College of Water Resources Science and Engineering, Taiyuan University of Technology, No.79 West Street Yingze, Taiyuan, 030024 China; 4 School of Environmental Science and Engineering, Southern University of Science and Technology, 1088 Xueyuan Avenue, Shenzhen, 518055 China

**Keywords:** Hydrology, Solid Earth sciences

## Abstract

Thermal conductivity is a basic parameter of soil heat transferring, playing an important role in many fields including groundwater withdrawal, ground source heat pump, and heat storage in soils. However, it usually requires a lot of time and efforts to obtain soil thermal conductivity. To conveniently obtain accurate soil thermal conductivity, a new model describes the relationship between soil thermal conductivity (*λ*) and degree of saturation (*S*_*r*_) was proposed in this study. Dry soil thermal conductivity (*λ*_*dry*_) and saturated soil thermal conductivity (*λ*_*sat*_) were described using a linear expression and a geometric mean model, respectively. A quadratic function with one constant was added to calculate *λ* beyond the lower *λ*_*dry*_ and upper *λ*_*sat*_ limit conditions. The proposed model is compared with five other frequently used models and measured data for 51 soil samples ranging from sand to silty clay loam. Results show that the proposed model match the measured data well. The proposed model can be used to determine soil thermal conductivity of a variety of soil textures over a wide range of water content.

## Introduction

As an important part of the earth's critical zone, soil layer is a channel that controls the material transformation and energy flow^1–3^. In recent years, with the rise of geothermal energy^4–6^, understanding the soil thermal conductivity (*λ*) is crucial for designing and optimizing engineering projects^7, 8^. *λ* is one of the important parameters describing the thermal properties of soil. It represents the ability of soil to conduct heat and controls the heat transfer process in soil, which is a key parameter for geothermal energy application^9, 10^. In geothermal energy development, *λ* significantly affects the heat transfer process between buried pipes and the surrounding soil^11^. Accurately measure *λ* is important for determining the optimal spacing between geothermal wells^12–14^ and design parameters for ground source heat pump systems^15, 16^.

Many parameters, such as water content (*θ*), mineral composition, texture, temperature, confining pressure, bulk density (*ρ*_*b*_), and porosity (*n*), may affect soil thermal conductivity *λ*^17–21^. Although significant progress has been achieved in measuring techniques (e.g., heat pulse method or heat plate method), direct measurement for various types of soils remains time consuming, labor intensive, expensive, and impractical for larger-scale applications^19, 22^. As a result, majority of studies have focused on developing models based on widely available soil properties^23–28^.

Existing soil thermal conductivity models can be classified into two types: theoretical models and empirical models^19, 29–31^. An accurate theoretical model which has been widely used was proposed by De Vries^32^, however, numerous parameters need to be chosen as input in the model^33, 34^. Based on a great deal of experimental data, Kersten^35^ established an empirical model requiring only one parameter, i.e., bulk density (*ρ*_*b*_). But it isn’t appropriate for calculating *λ* at lower water contents. Lu and Dong^26^ put forward a closed-form equation that incorporates the influence of various soil types and water content on thermal conductivity, specifically for ambient temperatures ranging from 20 to 25 °C. Building upon the Lu and Dong (2015) model^26^, Duc et al.^21^ utilized the data obtained from Yao et al.^20^ and Xu et al.^36^ to develop a calculation model for thermal conductivity in unsaturated soil. This enhanced model takes into consideration the effects of water content, temperature, and confining pressure. However, too many physical parameters limit the use of the model in practical engineering.

Woodside and Messmer^37^ proposed a geometric mean model to calculate saturated soil thermal conductivity (*λ*_*sat*_), which contributed to the development of empirical models. Johansen^38^ proposed a linear relationship to calculate *λ* between *λ*_*sat*_ calculated by the geometric mean model^37^ and *λ*_*dry*_ with the Kersten number (*K*_*e*_). However, the Johansen (1975) model^38^ cannot calculate *λ* with low saturation (0 < *S*_*r*_ < 0.05), and great deviation can be seen for fine-textured soils. Previous studies attempted to modify the expression of *K*_*e*_ (the Kersten number)^29, 39, 40^. Campbell^41^ introduced an empirical function with 5 parameters which describes the relationship between *λ* and volumetric water content (*θ*). The Campbell (1985) model^41^ has been widely used in the literature^42–44^. However, the parameters in the Campbell (1985) model are difficult to obtain and the function is not suitable for a large variety of soil textures^45^. Hansson et al.^46^ modified the Campbell (1985) model by replacing $\theta$ with $(\theta { + }F\theta_{i} )$ to better describe the dependence of *λ* on ice and water contents of frozen soils. Tien et al.^47^ proposed an improved thermal probe method for thermal conductivity measurement, and established the relationship of *λ* with clay dry density, water content, and sand or crushed granite based on the Campbell (1985) model. Li et al.^42^ found that the Campbell (1985) model was quite different from measured data and then revised the expressions of parameters of the model. Modified Campbell (1985) models are emerging, suggesting that a unified and universally applicable model has not been found to calculate soil thermal conductivity^19^. Xu et al.^48^ highlighted the need for correcting the Campbell (1985) model before its application. They noted that the model fails to adequately capture the variation pattern between thermal conductivity (*λ*) and water content (*θ*) in different soil types, particularly under conditions of low water content. As the underground soil temperature for thermal energy storage increases, the soil near the ground heat exchanger tends to experience drying^49, 50^, significantly affecting the heat transfer efficiency and thermal storage capacity. Studying λ at lower water content can provide a more realistic assessment of soil’s thermal conduction performance, helping us optimize the efficiency of geothermal heat pump systems.

With the continuous development of the model, although the physical mechanisms considered by the model are becoming more and more comprehensive, the increase in parameters increases the computational difficulty of the model, limits the use of the model in practical engineering, and improves the accuracy to a limited extent. The objective of this study is to develop a new model for calculating soil thermal conductivity based on the Campbell (1985) model. The new model aims to provide more accurate predictions of the variation of soil thermal conductivity with water content. In addition, it addresses the issue in existing models where the varying growth rates of soil thermal conductivity at low water content stages, due to variations in specific surface area among different soil textures, is not adequately addressed. This problem is successfully resolved in this study by introducing a simple parameter.

## Materials and methods

### Campbell (1985) model

The Campbell (1985) model is an empirical model developed by Campbell^41^ for calculating the soil thermal conductivity of silt, loam, and forest litter. The model can be expressed as:

1 $$\lambda = A + B\theta + (A - D)\exp \left[ { - (C\theta )^{4} } \right]$$

2 $$A = 0.65 - 0.78\rho_{b} + 0.6\rho_{b}^{2}$$

3 $$B = 1.06\rho_{b}$$

4 $$C = 1 + 2.6/m_{c}^{0.5}$$

5 $$D = 0.03 + 0.1\rho_{b}^{2}$$

where *θ* is the volumetric water content, cm^3^/cm^3^; *A*, *B*, *C*, and *D* are parameters related to soil physical properties, *m*_*c*_ is the soil clay content, %; *ρ*_*b*_ is soil bulk density, g/cm^3^.

### Proposed model

**Figure 1:**
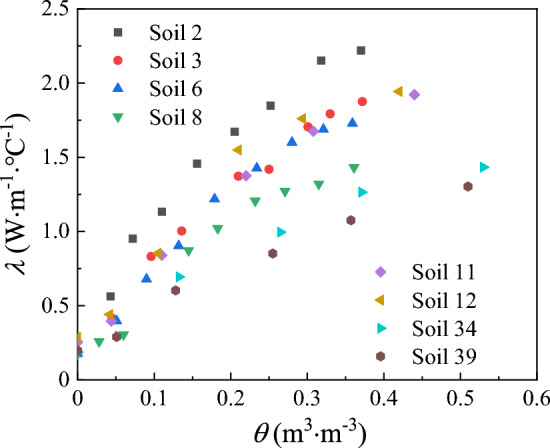
Relationship between soil thermal conductivity (*λ*) and water content (*θ*) for the 8 soils.

Previous studies show that values calculated by the Campbell (1985) model is much smaller than the measured values^42–44, 51^. Enhancing the Campbell (1985) model calculations requires calibration for different soils to obtain the model parameters which increases the complexity of the model. To improve the agreement between calculated and measured data, we modified the Campbell (1985) model by replacing *θ* with *S*_*r*_ and changing the exponential term. Upon examining the variation curve of soil thermal conductivity, it becomes evident that there is a linear increase in thermal conductivity with increasing water content within the range of 0–0.2 m^3^/m^3^ (Fig. 1). The propose model is split into three terms. The first two terms of the model consist of a linear function related to the saturation $\left( {P{ + }QS_{r}^{R} } \right)$. Since the thermal conductivity of different soil types increases with different rates. A parameter *R* was used to control the slope of the calculated *λ* plot at lower water content. Besides, a parameter *S* was introduced to control the calculations beyond the lower (*λ*_*dry*_) and upper (*λ*_*sat*_) limit conditions in the third item of the proposed model $(S\exp [S_{r} (1 - S_{r} )])$. The modified model is applicable for different types of soil, which can be described as following:

6 $$\lambda = P + QS_{r}^{R} + S\exp \left[ {S_{r} (1 - S_{r} )} \right]$$

7 $$P = \lambda_{dry} = a + bn$$

8 $$Q = \lambda_{sat} - \lambda_{dry}$$

9 $$S = c\left( {S_{r} - S_{r}^{2} } \right)$$

where *P*, *Q*, and *R* are related to physical properties of the soil (porosity, composition, and texture); *S* is related to *S*_*r*_; *S*_*r*_ is soil saturation, % and *c* is a constant; *λ*_*dry*_ is dry soil thermal conductivity, W/m °C and is saturated soil thermal conductivity, W/m °C.

Both the Tien (2005) model^47^ and the Li (2008) model^42^ are modifications of the Campbell model. However, the two models only adjust the coefficients of the Campbell model based on their measured data, without incorporating the variations in thermal conductivity. In contrast, the Johansen (1975) and Lu et al. (2007) models propose a linear relationship to calculate *λ* between *λ*_*sat*_, calculated using the geometric mean model^37^, and *λ*_*dry*_ with *K*_*e*_. Despite the significant improvement in model accuracy, the complex logarithmic or exponential formulations increase the computational difficulty of the two models. Many models have been developed to predict saturated soil thermal conductivity (*λ*_*sat*_) and soil solids thermal conductivity (*λ*_*s*_)^25, 52–55^. He et al.^30^ pointed out that the geometric mean model proposed by Woodside and Messmer^37^ is one of the most frequently used models. The model was described as following:

10 $$\lambda_{sat} = \lambda_{s}^{1 - n} \lambda_{w}^{n}$$

11 $$\lambda_{s} = \lambda_{q}^{q} \lambda_{0}^{1 - q}$$

where *n* is soil porosity, $\lambda_{w}$ (0.594 W/m °C) is the thermal conductivity of water, $\lambda_{0}$ is taken as 2.0 W/m °C for soils with *q* > 0.2, and 3.0 W/m °C for soils with *q* ≤ 0.2, *q* is the quartz content in the soil, $\lambda_{q}$ (7.7 W/m °C) is the thermal conductivity of quartz.

The Campbell (1985) model is used as a reference model. Two modified Campbell models by Tien et al.^47^ and Li et al.^42^, the Johansen (1975) model, and the Lu et al. (2007) model were also used as reference models to compare with the proposed model.

### Soil samples

The TEMPOS Thermal Properties Analyzer (TTPA) from METER Group (U.S.) which can read four different sensors was used to measure *λ*. TR-3 sensor (100 mm in length, 2.4 mm in diameter) was used to obtain *λ* within 1 min after inserting the sensor into soil. The values of *θ* were measured by Stevens Hydra probe II temperature and humidity probe.

We collected 8 soil samples from field. The 8 soil samples have different textures including sand, sandy clay loam, silty clay, loam, sandy loam, silt loam, and silt clay loam. Soil samples were air dried, ground, and sieved through a vibrating screen. Then the soil samples with different particle sizes were made according to the international standard for soil texture^56^. To reduce the impact of environment on the results, laboratory temperature was controlled at constant (20 °C) by air conditioner while measuring *λ*. Different soil water contents were obtained by adding a certain amount of water to the soil sample and thoroughly mixing the water and soil. Firstly, the 8 soil samples with different initial gravimetric water content of 0% (dry soil), 3%, 5%, 8%, 10%, 13%, 15%, 18%, and 20% were sealed and kept for 6 h, respectively. Secondly, the 8 soil samples with different gravimetric water contents were packed into sealed columns of *Φ*105 × 110 mm (diameter × height) and placed at least 12 h to ensure uniform distribution of water in soil. The porosity of soils was controlled by dry density which was govern through the height and mass of the soil in sealed columns. After setting for 12 h, *λ* was measured 3 times for each soil sample by a TR-3 sensor.

Measured datasets in the literature were also collected to verify the proposed model. The Tarnawski et al.^57^ dataset consisting of 39 soils from nine Canadian provinces and the Lu et al.^29^ dataset with 12 soils (10 from China, 2 from U.S.) were used. Detailed information on the texture and particle-size of all the 59 soils are listed in Table 1.

**Table 1:** Texture and particle-size distribution of the soils used.

Soils NO.	Soil name	Texture	Particle size distribution (mass %)			Bulk density	Porosity	Sources
			Clay	Silt	Sand			
1	XK-01	Sand	0.08	0.75	99.17	1.65	0.37	This study
2	XK-02	Sand	0.05	0.45	99.5	1.65	0.37	This study
3	XK-03	Sand	0.3	1.4	98.3	1.65	0.38	This study
4	XK-04	Sand	0.2	1.8	98	1.65	0.38	This study
5	XK-05	Sand	0.17	1.54	98.29	1.65	0.38	This study
6	XK-06	Clay loam	20.4	30	50.96	1.65	0.39	This study
7	XK-07	Sandy loam	0.8	10.3	92	1.65	0.37	This study
8	XK-08	Silty clay	26.67	73.33	0	1.65	0.40	This study
9	NS-05	Loamy sand	0.03	0.13	0.85	1.60	0.40	Tarnawski et al. (2014)
10	SK-04	Loamy sand	0.03	0.14	0.83	1.56	0.42	Tarnawski et al. (2014)
11	PE-01	Loam	0.08	0.42	0.5	1.48	0.44	Tarnawski et al. (2014)
12	NS-06	Sandy loam	0.06	0.38	0.56	1.32	0.51	Tarnawski et al. (2014)
13	NS-03	Sandy loam	0.05	0.37	0.57	1.61	0.4	Tarnawski et al. (2014)
14	SK-05	Sandy loam	0.05	0.28	0.68	1.47	0.45	Tarnawski et al. (2014)
15	NS-02	Sandy loam	0.05	0.34	0.61	1.49	0.45	Tarnawski et al. (2014)
16	MN-04	Loamy sand	0.03	0.15	0.81	1.43	0.47	Tarnawski et al. (2014)
17	SK-02	Sandy loam	0.06	0.27	0.67	1.49	0.45	Tarnawski et al. (2014)
18	NS-04	Sand	0	0	1	1.70	0.36	Tarnawski et al. (2014)
19	PE-02	Loam	0.09	0.39	0.51	1.54	0.42	Tarnawski et al. (2014)
20	NB-01	Silt loam	0.15	0.82	0.03	1.19	0.54	Tarnawski et al. (2014)
21	NB-02	Silt loam	0.17	0.83	0	1.12	0.56	Tarnawski et al. (2014)
22	NB-03	Silt loam	0.1	0.66	0.24	0.98	0.62	Tarnawski et al. (2014)
23	AB-01	Silt loam	0.1	0.52	0.38	1.19	0.55	Tarnawski et al. (2014)
24	PE-03	Loamy sand	0.03	0.14	0.83	1.57	0.41	Tarnawski et al. (2014)
25	NS-01	Silt loam	0.1	0.57	0.32	1.22	0.55	Tarnawski et al. (2014)
26	SK-01	Silt loam	0.26	0.74	0	1.59	0.41	Tarnawski et al. (2014)
27	QC-02	Loamy sand	0.03	0.17	0.79	1.40	0.48	Tarnawski et al. (2014)
28	ON-03	Loamy sand	0.04	0.26	0.71	1.46	0.46	Tarnawski et al. (2014)
29	NB-05	Silty clay loam	0.33	0.67	0	1.25	0.54	Tarnawski et al. (2014)
30	ON-04	Sand	0.01	0.1	0.89	1.68	0.39	Tarnawski et al. (2014)
31	ON-06	Loamy sand	0.02	0.14	0.84	1.53	0.44	Tarnawski et al. (2014)
32	MN-01	Silt loam	0.14	0.69	0.17	1.21	0.55	Tarnawski et al. (2014)
33	SK-03	Silt loam	0.15	0.83	0.02	1.27	0.53	Tarnawski et al. (2014)
34	BC-06	Silt loam	0.1	0.58	0.32	1.32	0.52	Tarnawski et al. (2014)
35	ON-05	Sandy loam	0.07	0.37	0.56	1.71	0.38	Tarnawski et al. (2014)
36	QC-01	Sand	0.02	0.05	0.93	1.55	0.43	Tarnawski et al. (2014)
37	NS-07	Silt loam	0.12	0.67	0.22	1.20	0.57	Tarnawski et al. (2014)
38	ON-01	Silt loam	0.08	0.56	0.37	1.54	0.43	Tarnawski et al. (2014)
39	BC-03	Silty clay loam	0.3	0.7	0	1.33	0.51	Tarnawski et al. (2014)
40	ON-07	Silt loam	0.14	0.54	0.32	1.52	0.45	Tarnawski et al. (2014)
41	MN-03	Silt loam	0.21	0.76	0.03	1.01	0.63	Tarnawski et al. (2014)
42	BC-01	Silty clay	0.42	0.58	0	1.34	0.51	Tarnawski et al. (2014)
43	MN-02	Silt loam	0.24	0.55	0.22	1.64	0.41	Tarnawski et al. (2014)
44	BC-02	Silty clay	0.42	0.58	0	1.36	0.5	Tarnawski et al. (2014)
45	ON-02	Silt loam	0.18	0.75	0.07	1.35	0.51	Tarnawski et al. (2014)
46	BC-04	Silt clay	0.41	0.59	0	1.34	0.52	Tarnawski et al. (2014)
47	BC-05	Clay	0.33	0.67	0	1.30	0.53	Tarnawski et al. (2014)
48	Lu-1	Sand	0.05	0.01	0.94	1.60	0.37	Lu et al. (2007)
49	Lu-2	Sand	0.06	0.01	0.93	1.60	0.37	Lu et al. (2007)
50	Lu-3	Sandy loam	0.12	0.21	0.67	1.39	0.46	Lu et al. (2007)
51	Lu-4	Loam	0.11	0.49	0.40	1.30	0.5	Lu et al. (2007)
52	Lu-5	Silt loam	0.22	0.51	0.27	1.33	0.51	Lu et al. (2007)
53	Lu-6	Silt loam	0.19	0.70	0.11	1.31	0.5	Lu et al. (2007)
54	Lu-7	Silty clay loam	0.27	0.54	0.19	1.30	0.5	Lu et al. (2007)
55	Lu-8	Silty clay loam	0.32	0.60	0.08	1.30	0.55	Lu et al. (2007)
56	Lu-9	Clay loam	0.30	0.38	0.32	1.29	0.55	Lu et al. (2007)
57	Lu-10	Silt loam	0.25	0.73	0.02	1.20	0.55	Lu et al. (2007)
58	Lu-11	Loam	0.09	0.41	0.50	1.38	0.51	Lu et al. (2007)
59	Lu-12	Sand	0.01	0.07	0.92	1.58	0.4	Lu et al. (2007)

### Model performance

Model performance was assessed by three goodness-of-fit parameters: (1) the root-mean square error (RMSE), (2) the average deviations (PBIAS), and (3) the Nash–Sutcliff Efficiency (NSE):

12 $$RMSE = \sqrt {\frac{1}{x}\sum\nolimits_{i = 1}^{x} {\left( {Y_{i}^{obs} - Y_{i}^{sim} } \right)^{2} } }$$

13 $$PBIAS = \sum\nolimits_{i = 1}^{x} {{{\left( {Y_{i}^{obs} - Y_{i}^{sim} } \right)} \mathord{\left/ {\vphantom {{\left( {Y_{i}^{obs} - Y_{i}^{sim} } \right)} {\sum\nolimits_{i = 1}^{x} {\left( {Y_{i}^{obs} } \right)} }}} \right. \kern-0pt} {\sum\nolimits_{i = 1}^{x} {\left( {Y_{i}^{obs} } \right)} }}}$$

14 $$NSE = 1 - \sum\nolimits_{i = 1}^{x} {{{\left( {Y_{i}^{obs} - Y_{i}^{sim} } \right)^{2} } \mathord{\left/ {\vphantom {{\left( {Y_{i}^{obs} - Y_{i}^{sim} } \right)^{2} } {\sum\nolimits_{i = 1}^{x} {\left( {Y_{i}^{obs} - \overline{{Y_{i}^{obs} }} } \right)} }}} \right. \kern-0pt} {\sum\nolimits_{i = 1}^{x} {\left( {Y_{i}^{obs} - \overline{{Y_{i}^{obs} }} } \right)} }}}^{2}$$

where *x* is the number of measurements; $Y_{i}^{obs}$ is the measured soil thermal conductivity; $Y_{i}^{sim}$ is the calculated soil thermal conductivity; $\overline{{Y_{i}^{obs} }}$ is the average of measured soil thermal conductivity.

The value of RMSE represents the efficiency of this model. The lower the value, the better the reliability of the model. PBIAS indicates the total amount of disparities between the calculated and measured values. PBIAS lower than 0.10 is desired, between 0.10 and 0.15 is reasonable, between 0.15 and 0.25 is satisfactory, and above 0.25 is not satisfied^58^. The value of NSE is less than 1, which is perfect above 0.75, good between 0.65 and 0.75, satisfactory between 0.5 and 0.65, and not satisfied below 0.5^59^.

## Results and discussion

### Relationship between soil thermal conductivity (*λ*) and volumetric water content (θ)

Figure 1 presents measured *λ* as a function of *θ* for the 8 soil samples with different textures. The influence of soil texture on the shape of *λ* curve has three distinct characteristics. First, the 8 soil samples with different textures have similar *λ* when the soil is dry, namely texture has little influence on *λ*_*dry*_. Second, sand (soils 2 and 3) has higher *λ* values than other soils and the greatest *λ* value appears in the soil which has the highest quartz content (*q*). Besides, at lower volumetric water contents, values of *λ* increased more gradually on fine-texture soils (soils 6, 11, 12, and 34); while *λ* values of sand (soils 2 and 3) and clay (soils 8 and 39) exhibit the fastest and the lowest rate of growth, respectively. The *θ* at which *λ* sharply increases is greater for clay than those for sand and fine-textured soils. This can be explained by the fact that clay has larger surface areas and more water is required before water bridges forms between soil solid particles^29, 60^.

The influence of *θ* on *λ* at room temperature can be explained by the process of substituting water for air between soil particles. Among solid, liquid, and air phases, the air phase has the lowest thermal conductivity (0.026 W/m °C), where water thermal conductivity (0.594 W/m °C) is 22 times greater than it. By analyzing the published datasets, four domains of soil water content were subdivided by Tarnawski and Gori^61^, i.e. residual, transitory meniscus, micro/macro porous capillary, and superfluous. At lower water content (residual water domain), water molecules adhere tightly to the surface of soil particles and the thickness of water film increases slowly with the increase of *θ*. Consequently, the soil thermal conductivity increases gradually but not significantly. With the further increase of *θ* (transitory meniscus, micro/macro porous capillary water domain), water bridges are constantly formed between soil particles, which leads to the increase of the contact area between soil particles and the rapid increase of soil thermal conductivity. This process does not stop until the air in the solid particle is completely replaced by water and the thermal conductivity is no longer increased (superfluous water domain).

### Determination of parameters

**Figure 2:**
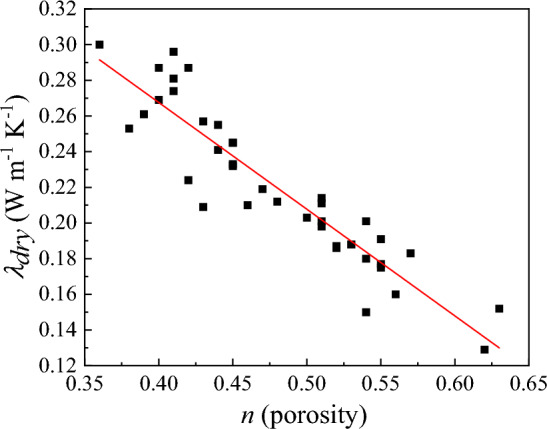
Relationship between dry soil thermal conductivity (*λ*_*dry*_) and porosity (*n*) based on published datasets from Tarnawski et al. (2014). The fitted linear equation was used in the proposed model to predict *λ*_*dry*_ from *n*.

At room temperature, most of the previous studies considered that porosity and quartz content are the main influencing factors of soil thermal conductivity^38, 62–64^. Chen^25^ also pointed out that the leading paths for thermal conduction between solid particles in a dry state are confined to particles contact points. In this paper, *n* is considered as the main factor affecting *λ*_*dry*_. Figure 2 shows the relationship between *λ*_*dry*_ and *n*. Clearly, *λ*_*dry*_ decreases linearly with *n*. Therefore, a simple linear formula similar to that of Lu et al.^29^, was developed to calculate *λ*_*dry*_. By fitting Eq. (7) to the data in Fig. 2, the calculated values of *a* and *b* were 0.51 and − 0.6, respectively.

**Figure 3:**
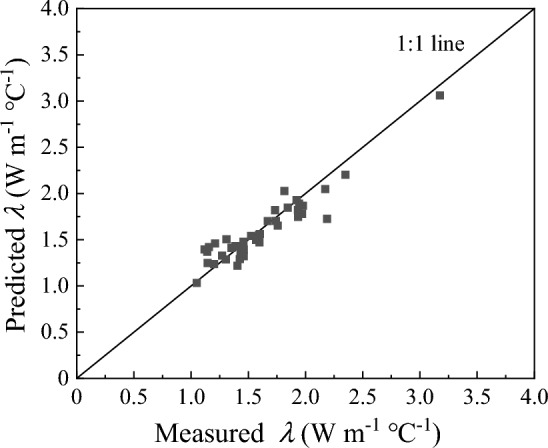
Comparison of predicted saturated soil thermal conductivity (*λ*_*sat*_) with measured data published datasets from Tarnawski et al. (2014).

Figure 3 shows that the geometric mean model proposed by Woodside and Messmer^37^ has a good performance in calculating the soil thermal conductivity (*λ*_*sat*_). He et al.^30^ verified the applicability of the geometric mean model, which also indicated a good performance. So, *λ*_*sat*_ was determined by Eqs. (10) and Eq. (11).

Tarnawski and Gori^61^ found that *λ* remained constant from dryness to a certain critical value of water content and it varied with soil texture. Clay and fine-textured soils have larger surface areas, so *λ* values increased more gently at low saturation while the *λ* values of sand had the fastest rate of growth. Johansen^38^ and Lu et al.^29^ also noticed this phenomenon and two different equations were used to calculate *K*_*e*_ from *S*_*r*_ for soils with different textures. In this study, we treated this by setting different *R* values. In Eq. (6), *R* controls the slope of the calculated *λ* plot at lower water content. Through trial calculations, a Microsoft Excel was used to assign different *R* values to different soil types so that the model calculation values agree well with the desired regular curve. The values of *R* are 1.2 for sand (soils 2 and 3), 1.5 is for the fine-textured soils (soils 6, 11, 12, and 34), and 2.0 for clay (soils 8, 39 and loamy clay).

**Figure 4:**
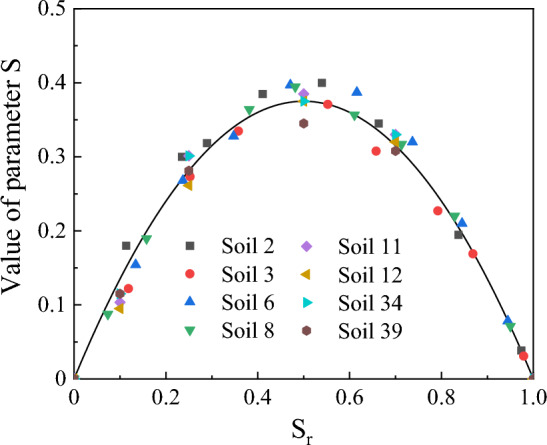
The dependence of parameter *S* on *S*_*r*_.

A 1stOpt program^65^ was used to find the optimized value of *S* using the Levenberg–Marquardt algorithm. Figure 3 presents the values of *S* as a function of *S*_*r*_ for the 8 soil samples. By fitting Eq. (9) to the optimized value of *S* in Fig. 4, the fitted value of constant c was 1.5.

Therefore, the proposed model can be described as followed:

15 $$P = \lambda_{dry} = 0.51 - 0.6n$$

16 $$Q = \lambda_{sat} - \lambda_{dry} = \left( {\lambda_{q}^{q} \lambda_{0}^{1 - q} } \right)^{1 - n} (0.594)^{n} - (0.51 - 0.6n)$$

17 $$S = 1.5\left( {S_{r} - S_{r}^{2} } \right)$$

### Model comparison for the eight typical soil samples

**Figure 5:**
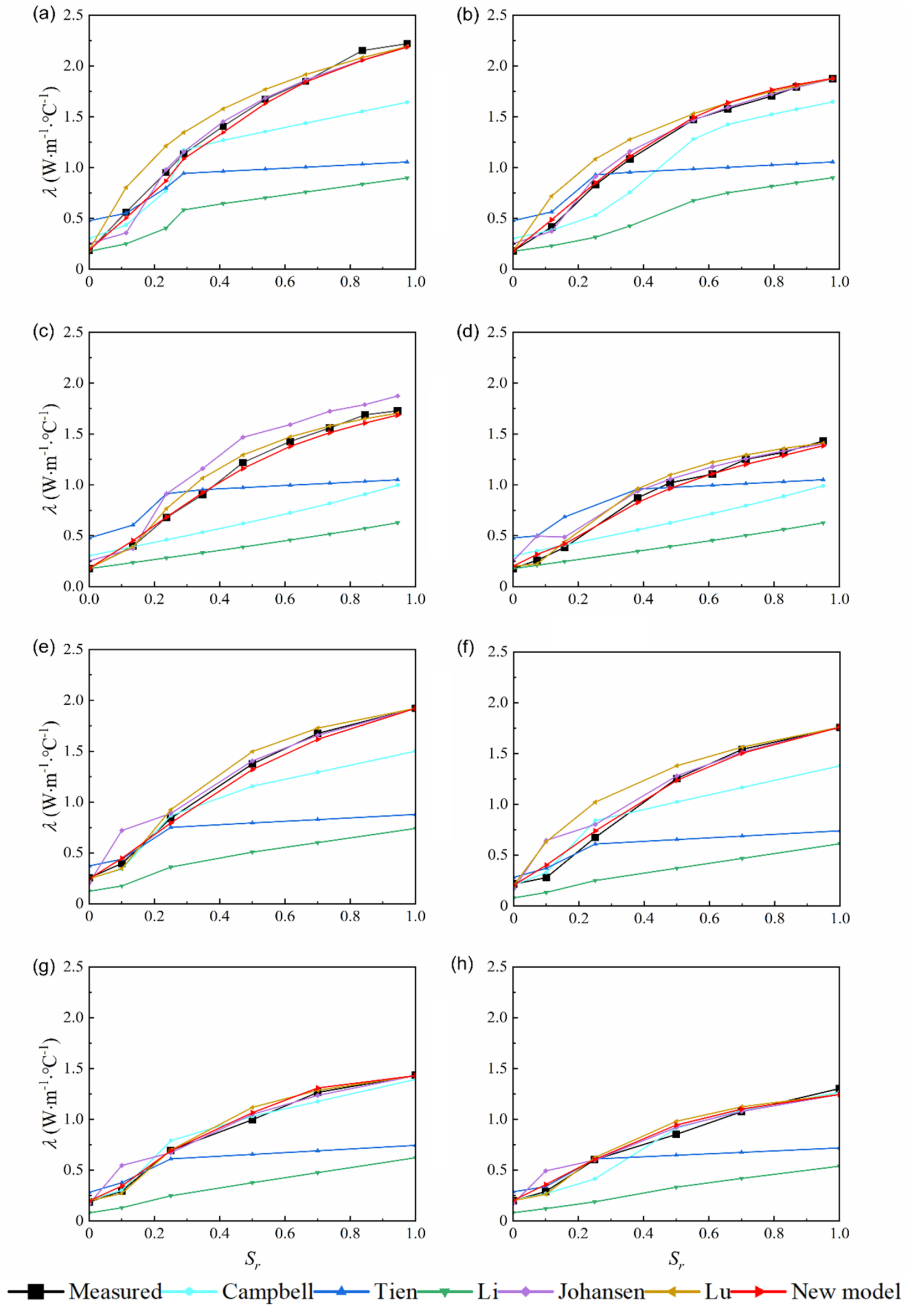
Comparison between calculated and measured soil thermal conductivity. (**a**) Sand (soil 2), (**b**) sand (soil 3), (**c**) sandy clay loam (soil 6), (**d**) silty clay (soil 8), (**e**) loam (soil 11), (**f**) sandy loam (soil 12), (**g**) silt loam (soil 34), and (**h**) silt clay loam (soil 39).

Figure 5 presents the comparison between the measured and calculated *λ* for the 8 typical soil samples. RMSE, PBIAS, and NSE of different models are listed in Table 2. The quartz contents of the 8 soils were not measured, so the measured *λ*_*sat*_ was used to estimate *λ* of the soils. Models of Campbell^41^, Tien et al.^47^, and Li et al.^42^ produced large deviations in the entire water content range. The Campbell (1985) model was able to provide more accurate calculations for silt loam and silt clay loam (Fig. 5g, h), but it was no longer suitable for other soils. The Campbell (1985) model underestimated the measured values for sand (Fig. 5a, b), loam (Fig. 5e), and sandy loam (Fig. 5f) under near-saturated conditions, while underestimated *λ* for sandy clay loam (Fig. 5c) and silty clay (Fig. 5d) in the entire water content range. For sand (Fig. 5a, soil 2), for example, the discrepancies of the Campbell (1985) model between measured and calculated *λ* values at *θ* = 0 is 0.1204 W/m °C, while it increased to 0.5763 W/m °C at *θ* = 20%. The Li et al. (2008) model, developed for loamy soil, underestimated *λ* for over the full texture ranges of soils. The Tien (2005) model generally overestimated *λ* at lower water contents and underestimated *λ* at higher water contents. In general, the Johansen (1975) model produced a good prediction of the thermal conductivity of the 8 soils, but there was a large error in predicting the thermal conductivity of dry soil. It overestimated the soil thermal conductivity of coarse sand and underestimated that of fine soils. For example, the measured values of dry soil thermal conductivity of soils 2, 12, and 39 are 0.21, 0.19, and 0.17 W/m °C, while the corresponding calculated values are 0.25, 0.21 and 0.20 W/m °C, respectively. The reason is that the Johansen's calculation formula of *λ*_*dry*_ has a close correlation with the dry bulk density of soil, while the dry bulk density of coarse sand is higher and the dry bulk density of fine soils are generally lower. This model also didn’t predict soil thermal conductivity well when the saturation is in the range of 0–5%. Compared with the Johansen (1975) model, the Lu et al. (2007) model produced a higher accuracy in predicting the thermal conductivity of fine-textured soils, but it was not as good as the Johansen (1975) model in predicting the thermal conductivity of coarse sand. In addition, the Lu et al. (2007) model obviously overestimates the soil thermal conductivity when soil saturation is in the range of 0.2–0.6. Those phenomena were not obvious in the new model. Therefore, we conclude that the new model, which provides the lower RMSE (0.04–0.06), PBIAS (-0.04–0.04), and the higher NSE (0.98–0.99), performs better than other models for all the tested soils. As shown in Table 2, the new model has the best performance on sand and the RMSE, PBIAS and NSE are 0.04 W/m °C, − 0.03 and 0.99, respectively. Although we did not obtain the exact quartz content, actual* λ* and quartz content measurements were listed in the paper^57^. Figure 5e–h show that the accuracy of the proposed model can be high.

**Table 2:** RMSE, PBIAS and NES of the models in predicting thermal conductivity of 8 soils.

Soil No	Campbell (1985)	Tien et al. (2005)	Li et al. (2008)	Johansen (1975)	Lu et al. (2007)	Proposed model
	RMSE/PBIAS/NES	RMSE/PBIAS/NES	RMSE/PBIAS/NES	RMSE/PBIAS/NES	RMSE/PBIAS/NES	RMSE/PBIAS/NES
2	0.341/0.181/0.735	0.678/0.356/− 0.05	0.875/0.567/− 0.745	0.082/0.015/0.985	0.156/− 0.107/0.944	0.056/0.035/0.993
3	0.213/0.138/0.868	0.517/0.265/0.219	0.721/0.53/− 0.517	0.047/− 0.024/0.994	0.150/− 0.103/0.935	0.04/− 0.026/0.995
6	0.553/0.412/− 0.044	0.424/0.18/0.384	0.795/0.633/− 1.159	0.077/− 0.032/0.980	0.070/− 0.036/0.983	0.047/0.02/0.993
8	0.337/0.275/0.455	0.253/0.012/0.693	0.566/0.547/− 0.543	0.104/− 0.070/0.949	0.069/− 0.048/0.977	0.049/0.007/0.989
11	0.25/0.164/0.84	0.601/0.372/0.08	0.774/0.612/− 0.527	0.136/− 0.055/0.953	0.067/− 0.033/0.988	0.043/0.018/0.995
12	0.247/0.136/0.83	0.597/0.415/0.01	0.66/0.55/− 0.209	0.160/− 0.075/0.929	0.209/− 0.140/0.879	0.059/− 0.022/0.99
34	0.058/− 0.005/0.984	0.397/0.311/0.272	0.564/0.604/-0.47	0.107/− 0.038/0.947	0.052/− 0.022/0.988	0.05/− 0.043/0.988
39	0.084/0.038/0.956	0.304/0.243/0.42	0.5/0.612/− 0.573	0.091/− 0.030/0.948	0.062/− 0.019/0.976	0.053/− 0.03/0.982

### Model evaluation and comparison using data in the literature

**Figure 6:**
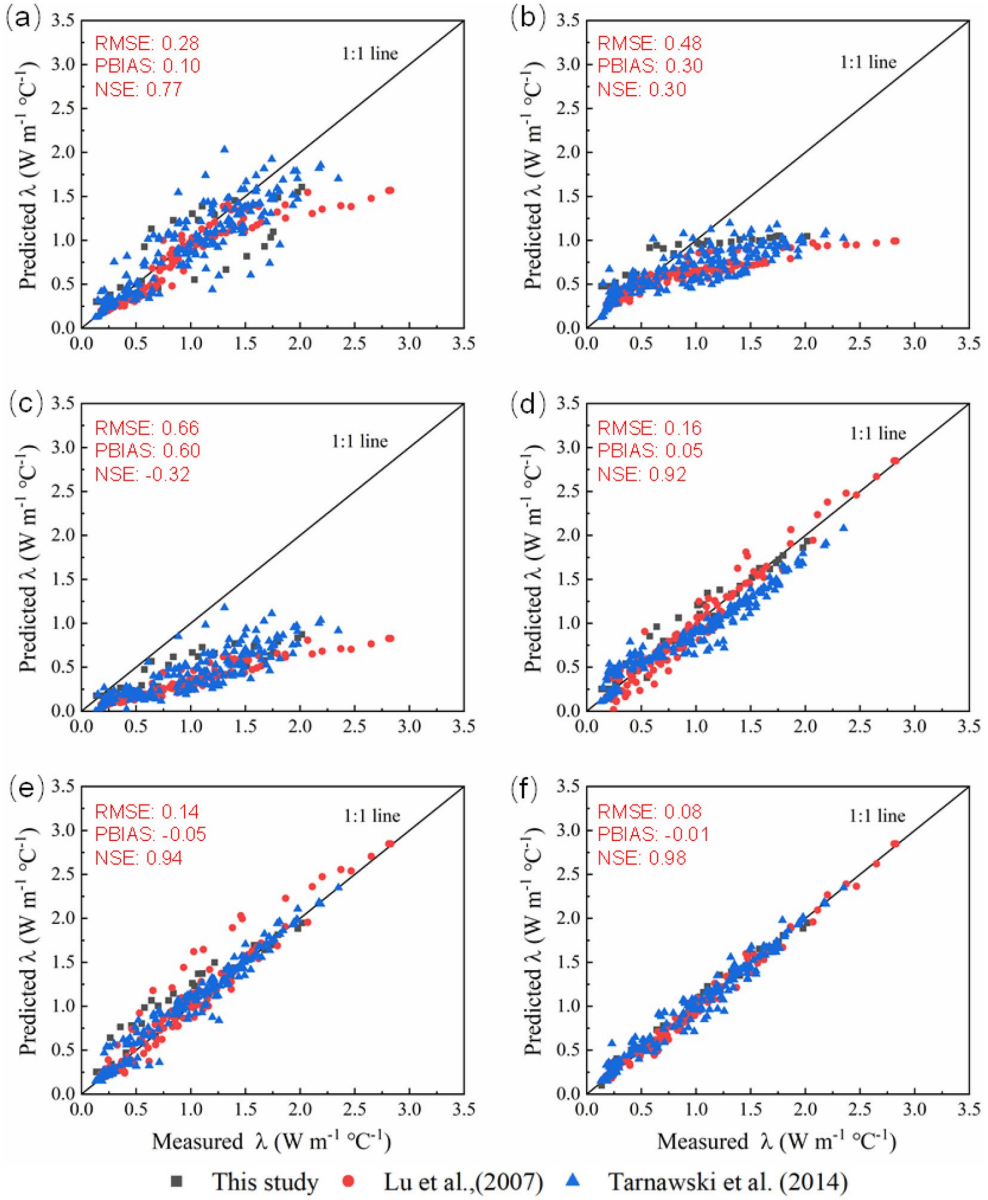
Comparison of predicted soil thermal conductivity (*λ*) from the six models with measured data. (**a**) The Campbell (1985) model, (**b**) the Tien et al. (2005) model, (**c**) the Li et al. (2008) model, (**d**) the Johansen (1975) model, (**e**) the Lu et al. (2007) model, and (**f**) the proposed model. Data source: this study (black square), Lu et al. (2007) (red dot), and Tarnawski et al. (2014) (blue triangle).

To further evaluate the accuracy of the proposed model, *λ* of soils with different textures and from different regions were used. Published datasets from Tarnawski et al.^57^ and Lu et al.^29^ and measured data in this study (soils 1, 4, 5, and 7) were used to evaluate the six models. Lu et al. did not measure the quartz content, either. So, it was assumed that the quartz content equals to the sand content^29^. The results are shown in Fig. 6a–f and RMSE, PBIAS and NSE of the 6 models are listed in Table 3. The calculated *λ* values from the proposed model agreed well with the measured *λ* for all the 51 soil samples, as indicated by the distribution of the data along the 1:1 line. The proposed model works best among the six models with the lowest RMSE (0.08), lowest PBIAS (− 0.01), and the highest NSE (0.98), followed by the Lu et al. (2007), Johansen (1975) and Campbell (1985) models. The Li et al. (2008) model and Tien et al. (2005) model are the worst for calculating *λ*.

**Table 3:** RMSE, PBIAS and NES of the models in predicting thermal conductivity of 51 soils from Tarnawski et al. (2014), Lu et al. (2007) and this study.

Soil No.	Campbell (1985)	Tien et al. (2005)	Li et al. (2008)	Johansen (1975)	Lu et al. (2007)	New model
	RMSE/PBIAS/NES	RMSE/PBIAS/NES	RMSE/PBIAS/NES	RMSE/PBIAS/NES	RMSE/PBIAS/NES	RMSE/PBIAS/NES
1	0.256/0.07/0.823	0.571/0.418/0.121	0.71/0.969/− 0.388	0.107/− 0.001/0.969	0.193/− 0.100/0.899	0.073/− 0.009/0.986
4	0.547/0.657/0.032	0.461/0.284/0.312	0.823/1.69/− 1.192	0.054/0.013/0.990	0.168/− 0.091/0.908	0.058/− 0.035/0.989
5	0.286/− 0.262/0.481	0.265/− 0.143/0.553	0.286/0.409/0.482	0.206/− 0.262/0.732	0.317/− 0.411/0.363	0.06/− 0.043/0.977
7	0.084/0.084/0.956	0.316/0.301/0.371	0.504/1.744/− 0.601	0.086/− 0.029/0.953	0.039/− 0.024/0.990	0.063/− 0.035/0.975
9	0.368/0.199/0.773	0.762/0.394/0.027	0.920/0.586/− 0.416	0.241/0.063/0.903	0.093/− 0.021/0.986	0.057/0.006/0.995
10	0.737/0.53/− 0.58	0.585/0.374/0.004	0.959/0.715/− 1.680	0.285/0.189/0.763	0.190/0.116/0.895	0.127/0.079/0.953
13	0.19/0.12/0.921	0.611/0.326/0.181	0.749/0.533/− 0.232	0.184/0.076/0.926	0.114/− 0.041/0.972	0.039/0.017/0.997
14	0.135/0.048/0.95	0.547/0.334/0.175	0.673/0.550/− 0.250	0.178/0.085/0.912	0.059/− 0.027/0.990	0.040/0.013/0.996
15	0.287/0.206/0.798	0.636/0.397/0.01	0.825/0.626/− 0.664	0.230/0.114/0.871	0.056/0.023/0.992	0.083/0.041/0.983
16	0.376/0.274/0.6	0.709/0.478/− 0.422	0.925/0.690/− 1.419	0.239/0.156/0.838	0.059/0.002/0.990	0.116/0.075/0.962
17	0.365/0.263/0.596	0.599/0.406/− 0.088	0.834/0.650/− 1.112	0.320/0.208/0.689	0.199/0.122/0.880	0.129/0.043/0.950
18	0.511/− 0.649/− 0.366	0.306/− 0.303/0.509	0.090/0.061/0.957	0.163/− 0.035/0.861	0.136/− 0.097/0.903	0.050/− 0.015/0.987
19	0.174/0.07/0.928	0.586/0.327/0.174	0.706/0.531/− 0.199	0.240/0.143/0.861	0.052/− 0.015/0.993	0.089/0.030/0.981
20	0.113/0.02/0.947	0.466/0.384/0.101	0.616/0.667/− 0.570	0.147/0.036/0.910	0.061/− 0.016/0.984	0.062/− 0.038/0.984
21	0.116/0.103/0.938	0.439/0.394/0.115	0.610/0.724/− 0.710	0.146/− 0.001/0.902	0.109/− 0.071/0.946	0.117/− 0.117/0.937
22	0.14/− 0.06/0.865	0.355/0.403/0.134	0.559/0.825/− 1.144	0.107/− 0.007/0.922	0.103/− 0.081/0.928	0.084/− 0.119/0.952
23	0.176/0.091/0.86	0.451/0.403/0.081	0.657/0.741/− 0.952	0.150/0.031/0.899	0.087/− 0.073/0.965	0.072/− 0.055/0.977
24	0.183/0.126/0.913	0.579/0.343/0.132	0.742/0.556/− 0.425	0.237/0.165/0.855	0.126/− 0.083/0.959	0.106/0.055/0.971
25	0.211/0.213/0.81	0.489/0.426/− 0.017	0.699/0.737/− 1.076	0.133/0.042/0.925	0.045/− 0.008/0.991	0.067/0.055/0.981
26	0.137/0.021/0.952	0.529/0.287/0.284	0.654/0.495/− 0.094	0.199/0.106/0.899	0.077/− 0.045/0.985	0.051/0.009/0.993
27	0.215/− 0.007/0.827	0.463/0.34/0.195	0.647/0.593/− 0.570	0.201/0.108/0.849	0.113/− 0.050/0.952	0.086/0.027/0.972
28	0.132/0.113/0.92	0.413/0.318/0.214	0.635/0.603/− 0.863	0.198/0.147/0.820	0.056/− 0.013/0.985	0.115/0.056/0.939
29	0.196/0.105/0.827	0.454/0.39/0.072	0.666/0.724/− 1.000	0.134/0.034/0.919	0.092/− 0.064/0.962	0.041/− 0.028/0.992
30	0.171/0.039/0.884	0.347/0.203/0.521	0.557/0.465/− 0.233	0.271/0.221/0.709	0.063/0.028/0.984	0.097/0.008/0.963
31	0.106/0.075/0.96	0.425/0.271/0.352	0.612/0.542/− 0.341	0.247/0.166/0.782	0.105/− 0.026/0.960	0.093/0.048/0.969
32	0.072/0.057/0.977	0.427/0.356/0.172	0.589/0.666/− 0.570	0.139/0.020/0.912	0.108/− 0.082/0.948	0.066/− 0.060/0.980
33	0.059/0.054/0.979	0.329/0.281/0.338	0.528/0.657/− 0.699	0.123/0.037/0.908	0.055/− 0.014/0.981	0.038/− 0.013/0.991
35	0.159/− 0.04/0.915	0.329/0.117/0.636	0.474/0.404/0.243	0.201/0.115/0.864	0.070/0.027/0.984	0.097/− 0.061/0.969
36	0.109/0.06/0.949	0.396/0.277/0.328	0.606/0.538/− 0.572	0.231/0.167/0.772	0.089/0.026/0.966	0.136/0.003/0.921
37	0.124/0.122/0.924	0.423/0.38/0.117	0.619/0.717/− 0.891	0.122/0.013/0.926	0.197/− 0.159/0.809	0.068/− 0.075/0.977
38	0.171/− 0.16/0.876	0.338/0.185/0.518	0.455/0.427/0.122	0.167/0.105/0.881	0.061/− 0.021/0.984	0.047/− 0.026/0.991
40	0.113/− 0.113/0.936	0.287/0.146/0.585	0.436/0.458/0.044	0.157/0.086/0.875	0.061/− 0.036/0.981	0.081/− 0.072/0.967
41	0.061/− 0.051/0.972	0.309/0.312/0.283	0.476/0.740/− 0.700	0.137/− 0.001/0.859	0.149/− 0.106/0.833	0.180/− 0.262/0.758
42	0.196/− 0.235/0.69	0.22/0.07/0.608	0.330/0.474/0.117	0.111/0.002/0.900	0.144/− 0.094/0.833	0.145/− 0.177/0.830
43	0.292/0.194/0.799	0.613/0.337/0.118	0.793/0.556/− 0.479	0.177/0.138/0.926	0.164/0.050/0.937	0.101/− 0.002/0.976
44	0.193/− 0.232/0.721	0.229/0.067/0.607	0.334/0.464/0.164	0.115/0.007/0.901	0.134/− 0.088/0.865	0.130/− 0.157/0.873
45	0.189/− 0.196/0.692	0.201/0.112/0.653	0.394/0.487/− 0.046	0.109/0.061/0.897	0.070/− 0.035/0.958	0.076/− 0.088/0.951
46	0.295/− 0.424/0.224	0.196/0.022/0.658	0.284/0.422/0.281	0.104/0.009/0.905	0.121/− 0.080/0.870	0.138/− 0.181/0.830
47	0.119/0.142/0.879	0.213/0.085/0.611	0.360/0.531/− 0.110	0.104/0.004/0.907	0.138/− 0.097/0.837	0.150/− 0.201/0.807
48	0.731/0.359/0.351	1.058/0.496/− 0.359	1.247/0.673/− 0.888	0.169/− 0.076/0.965	0.330/− 0.257/0.868	0.065/− 0.008/0.995
49	0.657/0.322/0.521	0.825/0.419/− 0.015	1.024/0.655/− 0.562	0.182/− 0.113/0.951	0.377/− 0.330/0.796	0.050/− 0.023/0.996
50	0.318/0.239/0.686	0.65/0.444/− 0.311	0.858/0.681/− 1.286	0.980/0.023/0.081	0.977/-0.044/0.085	0.055/0.031/0.991
51	0.14/0.148/0.887	0.392/0.355/0.117	0.608/0.688/− 1.123	0.099/0.040/0.943	0.087/− 0.006/0.957	0.043/0.003/0.989
52	0.082/0.061/0.939	0.262/0.22/0.379	0.495/0.632/− 1.225	0.102/0.067/0.905	0.043/0.034/0.983	0.050/− 0.026/0.977
53	0.104/0.085/0.937	0.347/0.284/0.293	0.551/0.646/− 0.786	0.108/0.030/0.931	0.047/0.003/0.987	0.052/− 0.024/0.984
54	0.087/0.098/0.941	0.278/0.189/0.389	0.485/0.647/− 0.852	0.080/− 0.057/0.949	0.084/− 0.104/0.944	0.037/− 0.018/0.989
55	0.042/− 0.018/0.988	0.295/0.219/0.419	0.475/0.598/− 0.508	0.086/− 0.035/0.950	0.054/− 0.058/0.980	0.039/− 0.006/0.990
56	0.093/0.102/0.943	0.318/0.254/0.335	0.515/0.65/− 0.75	0.147/0.156/0.857	0.104/0.126/0.928	0.076/0.051/0.962
57	0.115/0.131/0.907	0.363/0.353/0.065	0.586/0.717/− 1.431	0.154/0.153/0.831	0.096/0.105/0.935	0.060/0.021/0.974
58	0.159/0.136/0.89	0.42/0.335/0.237	0.625/0.649/− 0.687	0.143/0.130/0.912	0.110/0.092/0.947	0.114/0.110/0.944
59	0.266/0.074/0.812	0.534/0.314/0.244	0.703/0.551/− 0.313	0.152/0.089/0.939	0.137/− 0.074/0.950	0.102/0.039/0.973

In the process of model validation, we found that the Campbell (1985) model (RMSE/PBIAS/NSE = 0.28/0.10/0.77) was greatly affected by soil bulk density, and it could better predict *λ* within the bulk density range of 1.2–1.4 g cm^−3^. As shown in Fig. 6a, most of the symbols in the model are located below the 1:1 line, implying that the calculated values are smaller than the measured value, which is consistent with Zhao et al.^51^. The Li et al. (2008) model (RMSE/PBIAS/NSE = 0.66/0.60/− 0.32) and the Tien et al. (2005) model (RMSE/PBIAS/NSE = 0.48/0.30/0.30) modified from the Campbell (1985) model mainly developed for fine-textured soils and frozen soils perform the worst that their model calculations severely underestimated the actual soil thermal conductivity based on results in Fig. 6b, c. Their calculations show similar increase trend as they only changed the model coefficients and didn’t further adjust the model structure. Since the inaccurate calculation of *λ*_*dry*_, the Johansen (1975) model (RMSE/PBIAS/NSE = 0.16/0.05/0.92) overestimates the soil thermal conductivity at low water content and underestimates the soil thermal conductivity at high water content^29, 39, 51^ (Fig. 6d). The Lu et al. (2007) model (RMSE/PBIAS/NSE = 0.14/− 0.05/0.94) was improved with fitted parameters, however, it does not give satisfactory results for some soils at low water contents (Fig. 6e). Notably, the Lu et al. (2007) model improves the performance of the Johansen (1975) model at low water contents, especially on fine-textured soils. The NSE values of soil 10, 17 and 30 improve from 0.763, 0.689 and 0.709 to 0.895, 0.880 and 0.984, respectively (Table 3). In general, the normalized empirical model (Johansen (1975) model and Lu et al. (2007) model) perform better than the models modified from the Campbell (1985) model (Tien et al. (2005) model and Li et al. (2008) model).

Compared to the Campbell (1985) model, the new model introduces parameter *R* to control the shape of *λ* curve, while parameter *S* governs the calculated values between *λ*_*dry*_ and *λ*_*sat*_. In contrast to the Johansen model, the new model addresses the limitation of accurately estimating *λ* for *S*_*r*_ ranging from 0 to 0.05, and exhibits superior accuracy. When comparing to the Lu et al. (2007) model, both models demonstrate similar accuracy, but they address the issue of varying growth rates of thermal conductivity at low water content stages for different soil textures differently. Lu et al.^29^ employs a complex exponential equation to tackle this problem, whereas the new model resolves it by introducing a single parameter, *R*, which is derived from extensive analysis and synthesis of experimental data. It is widely recognized that thermal conductivity measurements are primarily conducted in field settings, where simpler models prove more advantageous for practical field work. Additionally, the new model achieves high accuracy and has been developed and verified using a diverse range of soil textures. The proposed model accurately estimates *λ* across the entire water content range.

## Conclusions

A new model has been developed to calculate soil thermal conductivity based on water content, utilizing only a small set of simple parameters including quartz content and porosity. The new model demonstrates excellent agreement with the measurement data across the entire water content range of 8 soil samples. Moreover, it exhibits strong consistency with soils of various textures, as documented in the literature. The new model creatively solves the problem of inaccurate calculation of soil thermal conductivity in the low water content stage of existing models. These findings highlight the robustness and versatility of the proposed model, making it a valuable tool for accurately estimating the thermal conductivity of diverse soil types. The new model can be applied to distributed hydrological models in the future and can also provide approximate soil thermophysical property parameters for the construction of ground source heat pump systems.

While the new model successfully addresses the challenge of accurately calculating *λ* for different soil textures at low water content, it is worth noting that the current introduction of empirical parameters (e.g., parameter *R*) lacks direct practical significance. To further improve the applicability of model, future research endeavors should focus on establishing meaningful relationships between these empirical parameters and essential soil physical properties, such as bulk density and porosity. By providing practical interpretations for these parameters, the model can be refined, expanding its applicability and contributing to a more comprehensive understanding of soil behavior. Ultimately, these efforts would enhance the accuracy of thermal conductivity calculations.

## Supplementary Information

Supplementary Information.

## Data Availability

All data generated or analysed during this study are included in this published article and its appendix files.
