# Supplementary material for: A new model to predict soil thermal conductivity
Source: Sci Rep. 2023 Jul 1;13:10684. doi: 10.1038/s41598-023-37413-5 (PMC10314946; doi:10.1038/s41598-023-37413-5)
Supplement: Supplementary file 1 — Supplementary Information. [file 41598_2023_37413_MOESM1_ESM.docx]

**Appendix**

**Model S1: Tien et al. (2005) model**

The suitable coefficients for bentonite were established by Tien et al. (2005) using the same procedure proposed by Campbell (1985) with experimental data.

$\lambda=F+G\theta+(F-H)\exp[-(I\theta)^{4.1}]$ (18)

$F=\text{0.55-0.78}\rho_{b}+0.6\rho_{b}^{2}$ (19)

$G=0.259\rho_{b}$ (20)

$H=14.5$ (21)

$I=-0.067+0.2\rho_{b}^{2}$ (22)

**Li et al. (2008) model**

Li et al. (2008) fond that the calculated value by the Campbell (1985) model was quite different from the measured data. Li et al. (2008) modified the coefficients *A*, *B*, and *D* of the Campbell (1985) model:

$A=0.115-0.78\rho_{b}+0.6\rho_{b}^{2}$ (23)

$B=0.715\rho_{b}$ (24)

$D=-0.096+0.1\rho_{b}^{2}$ (25)

**Johansen (1975) model**

Johansen (1975) proposed a normalized soil thermal conductivity model which included a geometric mean model and a formula related to soil bulk density to calculate the thermal conductivity of saturated soil and dry soil, respectively. The model can be written as (Johansen, 1975):

$\lambda=(\lambda_{sat}-\lambda_{dry})K_{e}+\lambda_{dry}$ (26)

$K_{e}=\left\{\begin{matrix} 0.7\log S_{r}+1.0,\text{ }S_{r}>0.05 \\ \log S_{r}+1.0\text{ , }S_{r}>0.1 \end{matrix}\right.$ (27)

$\lambda_{sat}=\lambda_{s}^{1-n}\lambda_{w}^{n}$ (28)

$\lambda_{s}=\lambda_{q}^{q}\lambda_{0}^{1-q}$ (29)

$\lambda_{dry}=\frac{0.135\rho_{b}+64.7}{2700-0.947\rho_{b}}$ (30)

where *n* is soil porosity, $\lambda_{w}$ (0.594 W m^−1^°C^−1^) is the thermal conductivity of water, $\lambda_{0}$ is taken as 2.0 W m^−1^°C^−1^ for soils with *q*> 0.2, and 3.0 W m^−1^°C^−1^ for soils with *q* ≤ 0.2, $\lambda_{q}$ (7.7 W m^−1^°C^−1^) is the thermal conductivity of quartz.

**Lu et al. (2007) model**

On the basis of the Johansen (1975) model, Lu et al. (2007) developed an improved model that describes the relationship between thermal conductivity and volumetric water content of soils. The calculation of dry soil thermal conductivity is also improved by using a simpler linear formula. The Lu et al. (2007) model can be written as:

$\lambda=(\lambda_{sat}-\lambda_{dry})K_{e}+\lambda_{dry}$ (31)

$K_{e}=\exp(\alpha(1-S_{r}^{\alpha-1.33}))$ (32)

$\lambda_{dry}=-0.56n+0.51$ (33)

where α is soil texture-dependent parameter, α = 0.96 for coarse sand and 0.27 for fine-textured soils. The *λ_sat_* was calculated with Eq.10,11).
